# Analysis of criteria for choosing drug treatment strategies in allergic rhinitis

**DOI:** 10.3389/fphar.2024.1340554

**Published:** 2024-10-01

**Authors:** Damian Grzegorzewski, Marharyta Sobczak, Michał Tołkacz, Rafał Pawliczak

**Affiliations:** ^1^ Aurovitas Pharma Poland Ltd., Warsaw, Poland; ^2^ Department of Immunopathology, Medical Faculty, Medical University of Lodz, Lodz, Poland; ^3^ Quality Audit House Ltd., Lodz, Poland

**Keywords:** allergic rhinitis, treatment, decision-making, cost analysis, allergic rhinitis therapy

## Abstract

**Background:**

Allergic rhinitis (AR) is the most common type of rhinitis, the treatment of which relies on relieving symptoms. Therefore, we aimed to assess the criteria that influence doctors’ decision-making in the process of drug selection for the treatment of allergic rhinitis based on quantitative, qualitative, and cost analyses.

**Methods:**

We conducted a survey study with the participation of 300 allergologists. A self-developed questionnaire was presented during a computer-assisted telephone interview (CATI) according to

standard procedures. The contingency table underwent statistical analysis using the chi-square test with Cramer’s V. Results were considered statistically significant at *p* < 0.05.

**Results:**

Our analyses showed that doctors most often prescribe intranasal glucocorticoids and oral antihistamines to treat allergic rhinitis in patients of all ages. The most common factor that affects the decision-making related to AR treatment was the efficiency of the drug. We found a significant relationship between factors and the main workplace (X-squared = 122.81, df = 90, *p*-value = 0.0123, Cramer’s V = 0.1787216), as well as voivodeship of the main workplace (X-squared = 440.75, df = 270, *p*-value = 2.378e-10, Cramer’s V = 0.1954731). In our study, respondents claimed that patients are willing to pay 31–50 PLN (∼€7- €11) monthly for the treatment of mild and moderate forms of AR, while they were willing to pay 51–100 PLN (∼€11–€22) for treatment of the severe AR form.

**Conclusion:**

Our study confirms that the management of AR should be focused on the patient. One of the most important factors in choosing a drug is its effectiveness. Moreover, an important factor in the effective treatment of AR is the financial issue; as shown in our analysis, AR treatment costs can be a significant burden, especially for less wealthy citizens in Poland.

## 1 Introduction

Rhinitis is a common condition characterized by inflammation of the nasal mucosa. In addition to the classic symptoms, such as sneezing, nasal itching, rhinorrhea, and nasal congestion, symptoms related to the ears, eyes, and throat are also present. The most common type of rhinitis is allergic rhinitis (AR), which is mainly caused by the IgE-mediated immune response triggered by different allergens. Activation of the immune response leads to the production and release of inflammatory mediators and the activation and migration of different inflammatory cells, such as macrophages, mast cells, eosinophils, CD4-positive T cells, and B cells, to the nasal mucosa. Moreover, two types of AR occur during a specific season and the year: seasonal and perennial, respectively. However, not all patients can be classified as having the above types of AR. Consequently, AR is described based on severity as mild, moderate, and severe and based on symptom duration as intermittent and persistent (Small et al., 2018; Skoner, 2001). In addition to allergens, climate change may be one of the risk factors of AR because it may prolong the pollen season. Environmental factors may also have a negative influence on AR, e.g., air pollution may enhance the prevalence of AR (Zhang et al., 2021).

In 1999, during a WHO workshop, ARIA (Allergic Rhinitis and its Impact on Asthma) guidelines were developed, which were based on evidence and proposed the novel classification of AR (Euforea, 2023). However, recommendations for AR treatment were presented and updated in 2010 (Brożek et al., 2010). The choice of treatment for AR depends on different factors, e.g., age, control of AR, prominent symptoms and their severity, patient preferences, comorbidities, and cost of treatment (Meltzer, 2013). MASK-rhinitis (MACVIA-ARIA Sentinel NetworK for allergic rhinitis), developed by the European Innovation Partnership on Active and Healthy Aging, is a new system that not only focuses on diagnosing, stratifying, and treating patients with AR but also on assessing the efficacy of this treatment (Bousquet et al., 2015). Symptom relief is the aim of AR treatment, which includes different options, such as saline nasal irrigation, avoidance agents, intranasal corticosteroids, and oral antihistamines, as well as a combination of nasal antihistamines and corticosteroids, allergen immunotherapy, and leukotriene receptor antagonists, and, less frequently, oral corticosteroids and decongestants (Small et al., 2018). However, one of the most effective treatment options is allergen immunotherapy. Therefore, based on the 2019 ARIA Care pathways, clinicians should consider implementing this therapy in patients with AR. Allergen immunotherapy causes long-lasting positive effects through deep changes in the expression of genes and proteins in allergen-specific T cells in addition to the expression of proteins in plasma cells in nasal tissue (Zhang et al., 2021). However, the problem with this therapy is the long-term treatment time. In addition, there are many challenges, such as low patient compliance, high cost, sporadic severe side effects, and worldwide standardization (Meng et al., 2020).

Despite pharmacotherapy, a critical factor in the treatment of AR is patient knowledge, as not informing patients about the different treatment options can cause low adherence to doctor recommendations. Patients should be informed about the route of administration, the risks and benefits of existing treatments, and the drug’s mechanism of action, which may improve their regularity in taking treatments. Moreover, patients may be involved in doctors’ decision-making (Meng et al., 2020). Therefore, in the present study, we aimed to assess the criteria that influence doctors’ decision-making when selecting a drug for the treatment of allergic rhinitis based on quantitative, qualitative, and cost analyses.

## 2 Materials and methods

### 2.1 Study design and participants

According to Article 2 (Euforea, 2023) of Regulation (EU) No 536/2014 of the European Parliament and of the Council of 16 April 2014 on clinical trials on medicinal products for human use and repealing Directive 2001/20/EC (European Union, 2014), this study was non-interventional. Therefore, local bioethical committee approval was not required for this study.

We developed an in-house questionnaire to collect the answers to the questions mentioned in the *Introduction*. The questionnaire was pretested on a group of 25 allergy board-certified physicians randomly chosen from our database. Furthermore, corrections were made according to the data obtained from the pretest group. Therefore, the corrected questionnaire (Supplementary Material) was administered during the computer-assisted telephone interview (CATI) according to the standard procedures. The CATI was performed by an independent contractor: Quality Audit House, Lodz, Poland. The results were subjected to statistical analysis. A total of 300 allergy-board-certified physicians took part in the study.

### 2.2 Data processing

After data collection, we categorized data from open-ended questions, in which participants named the three most important factors in choosing a drug and the three most important sources of information. For the first open-ended question, answers were categorized as follows (other answers were not categorized):

• “Efficiency”: “efficiency” and “effectiveness.”
• “Occurrence of side effects”: “occurrence of side effects,” “no side effects,” “no complications,” “risk of complications,” and “long-term effects of treatment.”
• “Several forms of the drug adapted to the needs of different patients”: “several forms of the drug,” “form,” “form of the drug,” and “form of administration.”
• “Symptoms of the disease and their severity”: “symptoms of the disease and their severity,” “intensity of symptoms,” “severity,” “nasal congestion severity (AR),” “clinical condition of the patient,” “exacerbations,” and “stage of the disease.”
• “Sedative effect”: “sedative effect” and “effects on the central nervous system.”
• “Comfort/ease of use”: “comfort/ease of use,” “whether the patient can handle the drug,” “ease of administration,” and “method of administration.”
• “Patient preferences”: “patient preferences” and “what the patient prefers to be treated.”
• “Refund”: “refund” and “no refund.”
• “Interactions with other drugs”: “interactions with other drugs” and “other medications the patient is taking.”
• “Length of illness”: “length of illness” and “length of symptoms.”
• “Patient’s experience with the drug”: “the patient’s experience with the drug” and “the patient’s feelings after using the drug.”

For the second open-ended question, answers were categorized as follows (other answers were not categorized):

• “Medical literature”: “medical literature,” “books,” “medical magazines,” “popular science magazines,” “press,” “medical articles,” “literature,” “textbooks,” “scientific studies,” “medical newspapers,” “meta-analyses,” “scientific research,” “pubmed,” “internet (medical articles about research),” and “articles on the Internet.”
• “Information at conferences/trainings”: “information at conferences/trainings,” “symposia,” “congresses,” “conventions,” “meetings,” “foreign conferences,” “conferences for specialists,” “training,” “internet-training,” “online training,” and “training with educational points.”
• “Internet”: “internet,” “internet portals,” “specialized websites,” “databases,” “websites,” “chpl,” “RED,” “mp.pl,” and “pharmindex.”
• “Lectures”: “lectures,” “video lectures with experts,” “podcasts,” and “webinars.”
• “Leaflets/printed brochures”: “leaflets/printed brochures,” “leaflets,” “printed brochures,” and “brochures.”
• “E-mailings/newsletters”: “e-mailings/newsletters” and “newsletters.”
• “Visits of medical representatives”: “visits of medical representatives” and “visits of representatives.”

### 2.3 Statistical analysis

In order to check the distribution between the examined criteria (such as gender, main workplace, size of the town of the main workplace, voivodeship of the main workplace, and work experience after board certification) and the answers to the survey questions, we used cross-tabulations. In order to check whether there is a statistically significant relationship between two categorical variables, we used the chi-square test with Cramer’s V to measure the association between these variables. Results were considered statistically significant at *p* < 0.05. Statistical analysis of the data was carried out in R (version 4.3.0).

## 3 Results

### 3.1 Participants

This study was conducted among 300 allergologists from Poland. Among the participants, 74.7% were women, 47.7% of whom worked in public specialist clinics and 24.4% in private specialist clinics. In turn, 80.0% of the participants worked in cities with >100 thousand inhabitants. A total of 14.7% and 13.3% of respondents worked in Mazowieckie Voivodeship and Małopolskie Voivodeship, respectively. Moreover, 37.7% of participants have 21–30 years of work experience (after board certification). Detailed characteristics of doctors participating in this study are shown in Table 1.

**TABLE 1:** Characteristics of participants.

Characteristics		n (%)
Total		300 (100%)
Gender	Female physician	224 (74.7%)
	Male physician	76 (25.3%)
Main workplace	Private primary care clinic	12 (4.0%)
	Private specialist clinic	73 (24.4%)
	Private practice	40 (13.4%)
	Public primary care clinic	5 (1.7%)
	Public specialist clinic	143 (47.7%)
	Another place	27 (9.0%)
Place of the main workplace—size of town	City <50 thousand inhabitants	54 (18.0%)
	City 50–100 thousand inhabitants	66 (22.0%)
	City >100 thousand inhabitants	180 (80.0%)
Place of main workplace—voivodeship	Dolnośląskie Voivodeship	18 (6.0%)
	Kujawsko-Pomorskie Voivodeship	10 (3.3%)
	Lubelskie Voivodeship	18 (6.0%)
	Lubuskie Voivodeship	4 (1.3%)
	Łódzkie Voivodeship	34 (11.3%)
	Małopolskie Voivodeship	40 (13.3%)
	Mazowieckie Voivodeship	44 (14.7%)
	Opolskie Voivodeship	10 (3.3%)
	Podkarpackie Voivodeship	10 (3.3%)
	Podlaskie Voivodeship	28 (9.3%)
	Pomorskie Voivodeship	18 (6.0%)
	Śląskie Voivodeship	22 (7.3%)
	Świętokrzyskie Voivodeship	18 (6.0%)
	Warmińsko-Mazurskie Voivodeship	6 (2.0%)
	Wielkopolskie Voivodeship	14 (4.7%)
	Zachodniopomorskie Voivodeship	6 (2.0%)
Work experience (after board certification)	≤10 years	41 (13.7%)
	11–20 years	70 (23.3%)
	21–30 years	113 (37.7%)
	≥31 years	74 (24.7%)
	Undefined	2 (0.7%)

### 3.2 Overall results of the questionnaire

**FIGURE 1:**
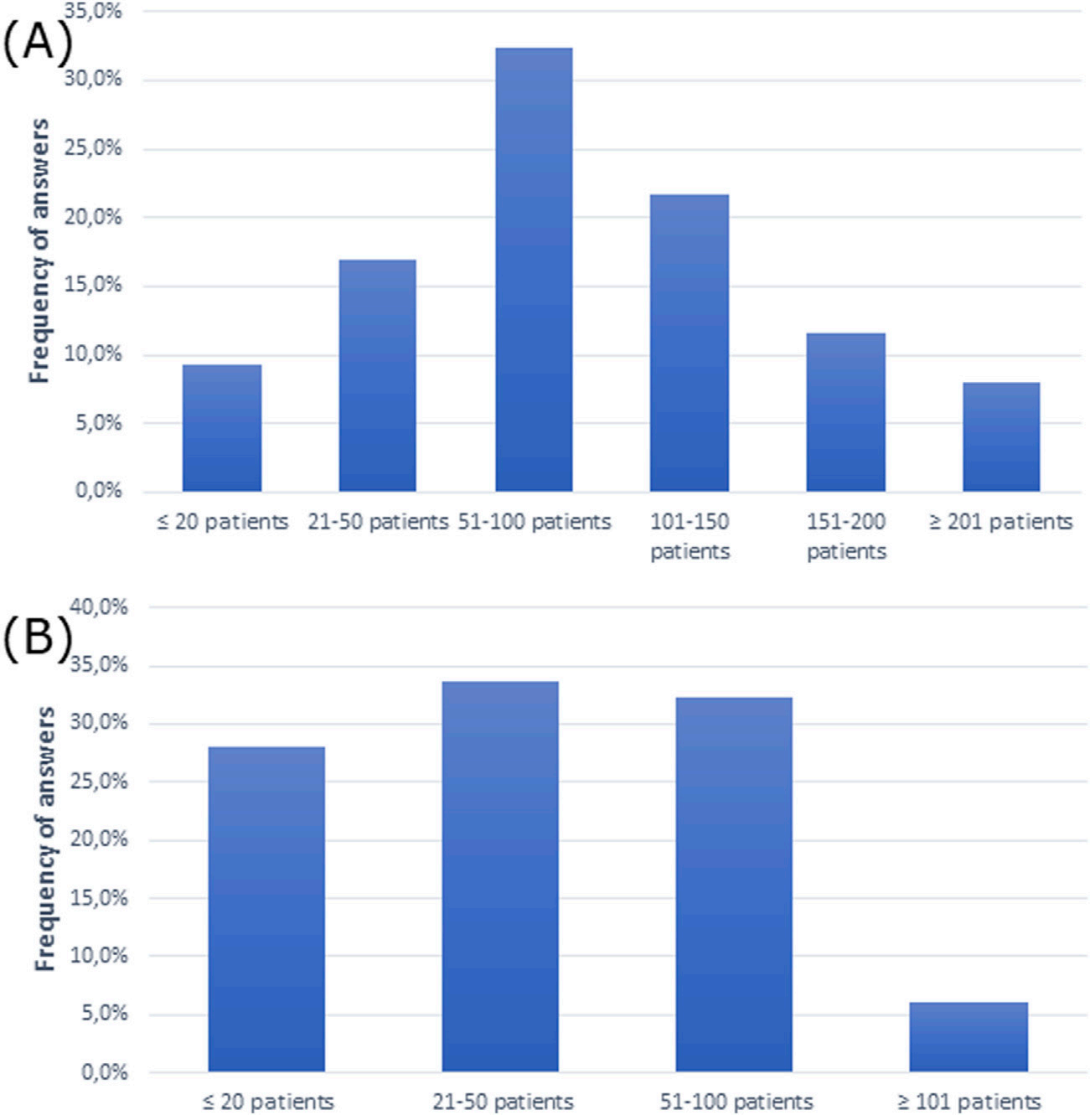
Average number of **(A)** patients and **(B)** patients with allergic rhinitis admitted weekly.

The questionnaire showed that the majority of participants had 51–100 patients per week and 21–50 patients per week with AR (Figures 1A, B).

**FIGURE 2:**
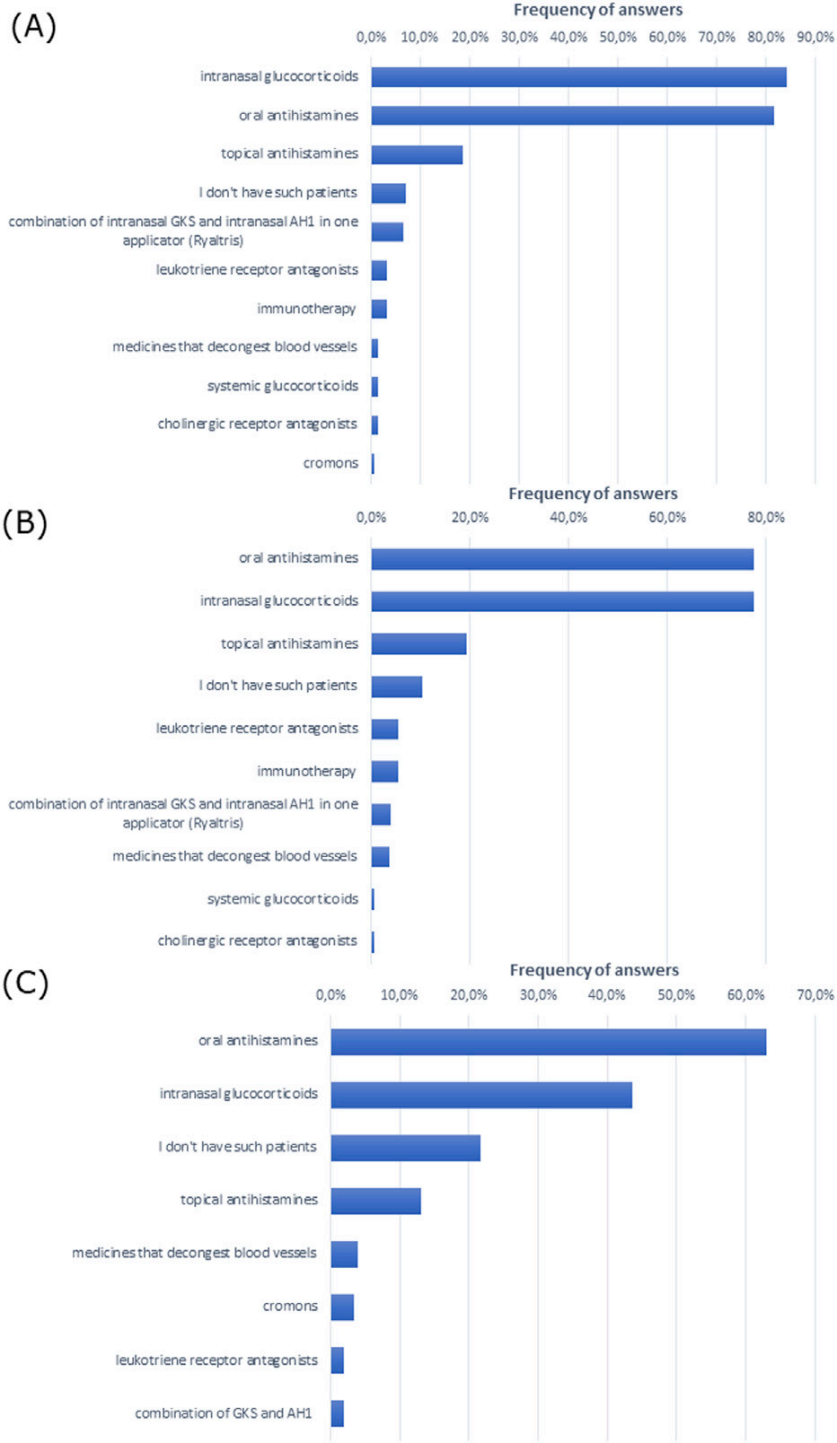
Most frequently prescribed treatment in **(A)** adult patients with allergic rhinitis (over 18 years of age), **(B)** patients aged 5–18 years, and **(C)** children up to 5 years of age (multiple choice).

To treat allergic rhinitis, doctors most often prescribed intranasal glucocorticoids and oral antihistamines in patients of all ages (Figures 2A–C). Notably, intranasal glucocorticoids were most commonly administered in the group of patients over 18 years of age, while oral antihistamines were most commonly administered in children up to 5 years of age.

**FIGURE 3:**
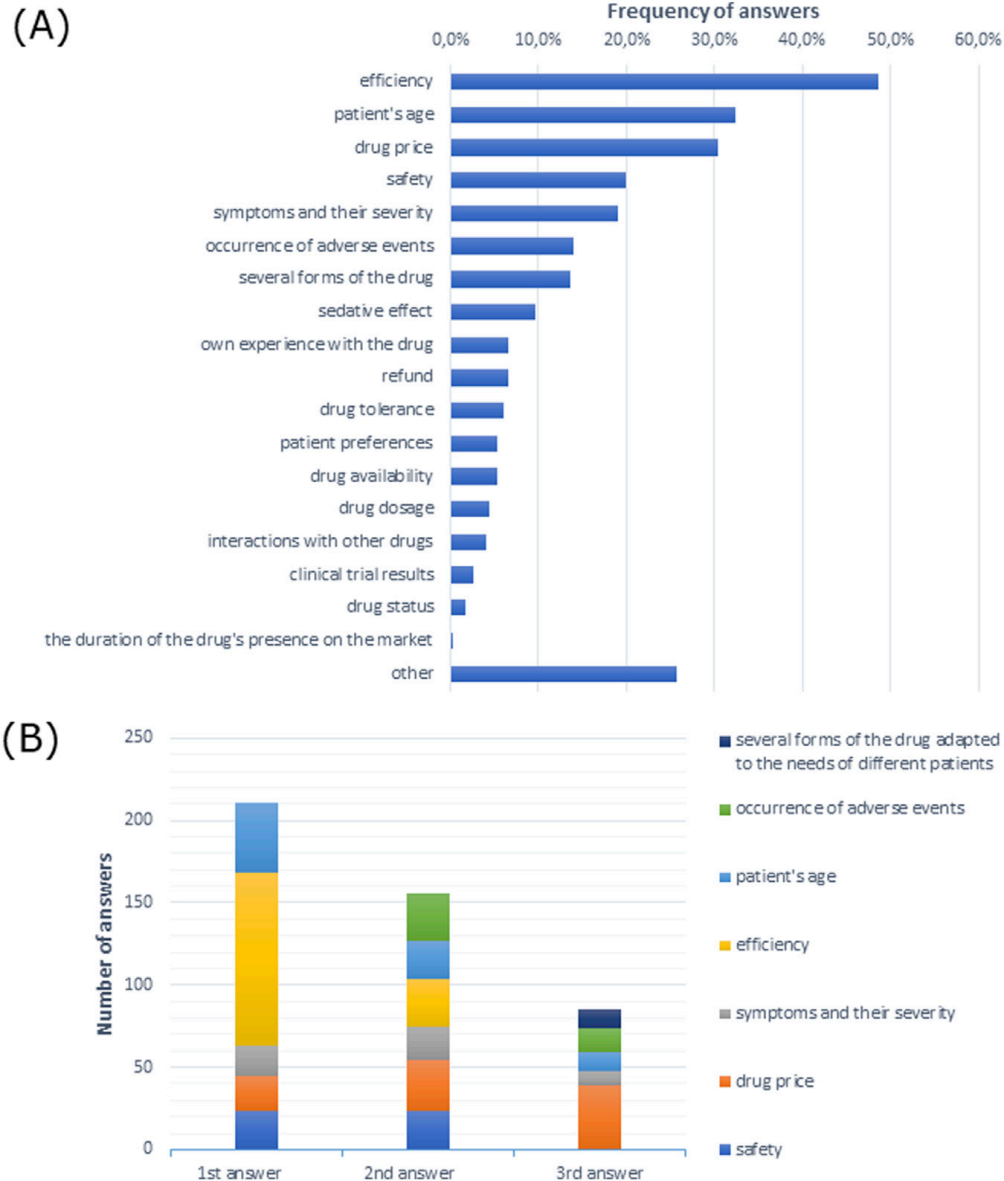
Factors indicated by allergologists influencing the choice of medication for the treatment of allergic rhinitis **(A)** in multiple-choice and **(B)** the most frequently indicated of the three most important factors justifying the choice of treatment presented in the order given by respondents.

Drug efficiency was the most commonly indicated factor influencing drug choice for the treatment of allergic rhinitis among participating doctors (Figure 3A). Similarly, efficiency was the most frequently indicated first response and drug price was the most frequently indicated second and third responses (Figure 3B).

**FIGURE 4:**
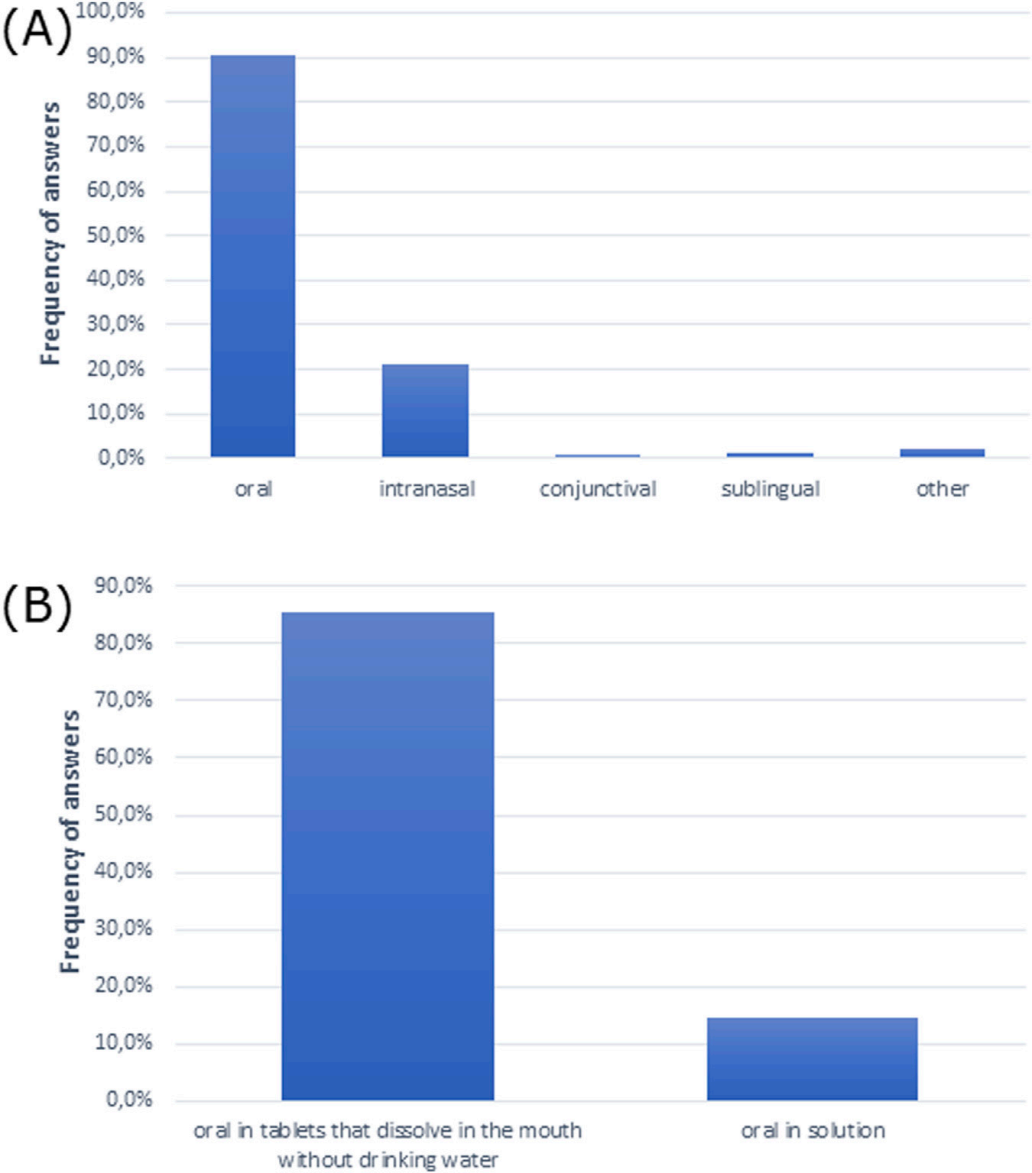
Routes of drug administration preferred by patients **(A)** overall (multiple choice) and **(B)** for oral drugs.

The oral route was the most common and preferred route of drug administration for patients. Moreover, patients opted for the intake of mouth-dissolving tablets without drinking water over the oral drugs in solution (Figures 4A, B).

**FIGURE 5:**
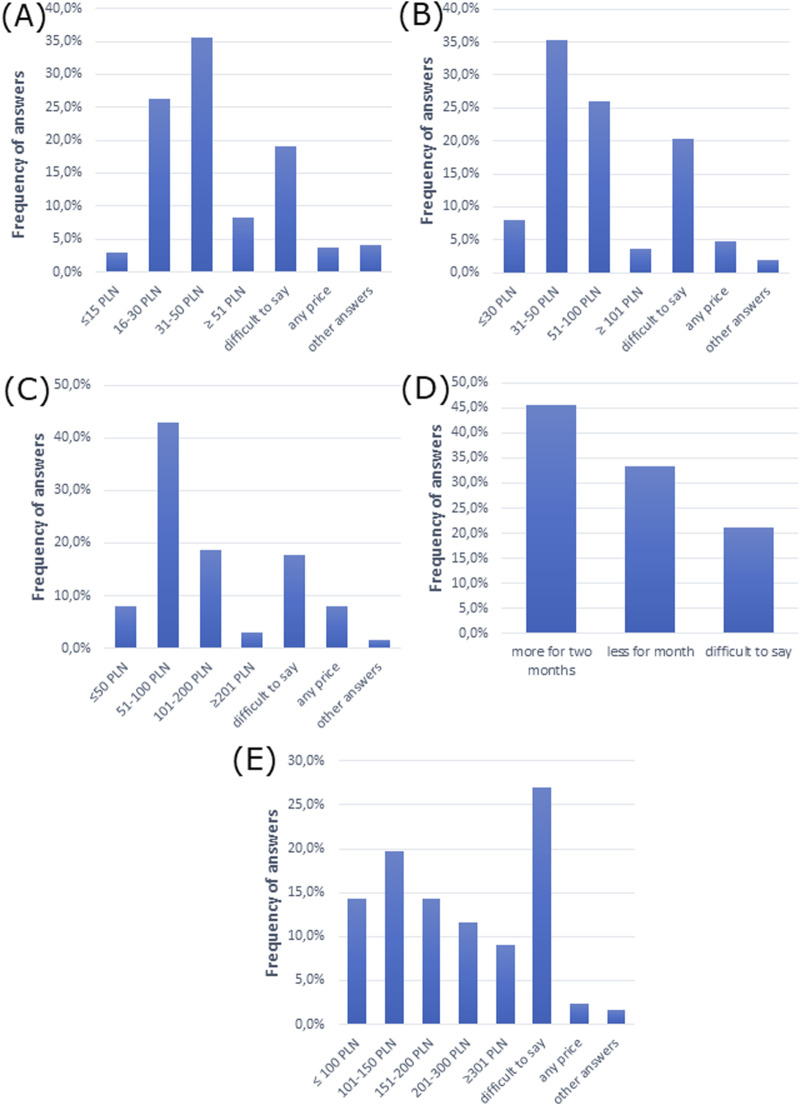
Cost analysis of allergic rhinitis treatment. The cost patients are willing to pay for monthly therapy of allergic rhinitis in **(A)** mild form, **(B)** moderate form, and **(C)** severe form. **(D)** Patient choice of drug comparing a cheaper drug for 1 month and a more expensive drug for 2 months of therapy. **(E)** The amount that patients are willing to spend per month on all medications (for all ailments).

We then analyzed the costs of treatment for allergic rhinitis. The cost of treatment was 31–50 PLN (∼€7–€11), which patients were willing to pay for monthly therapy of allergic rhinitis in mild and moderate forms, while 51–100 PLN (∼€11–€22) was the cost they were willing to pay for treatment of the severe form, according to doctors’ opinion (Figures 5A–C). Moreover, the majority of doctors claimed that patients selected more expensive drugs for 2 months of therapy than cheaper drugs for 1 month of therapy (Figure 5D). However, the majority of participants had difficulty pointing out how much patients would be willing to spend per month on all medications for all ailments (Figure 5E).

**FIGURE 6:**
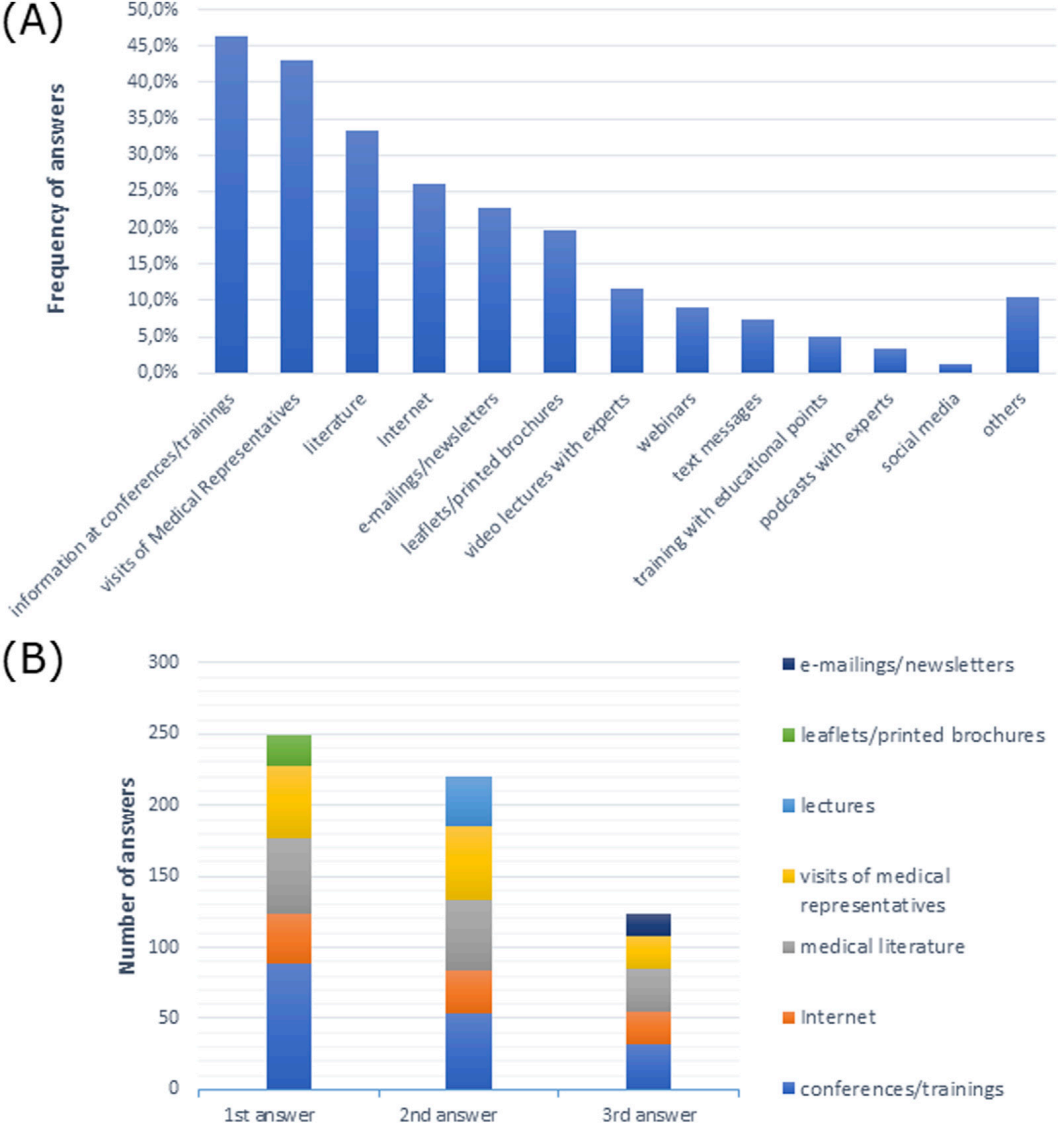
Sources of information preferred by doctors **(A)** in multiple-choice, and **(B)** most frequently indicated among the three most preferred sources of information in order of response.

Doctors most frequently indicated that they preferred conferences/trainings and visits from medical representatives as sources of information (Figure 6A). Conferences/trainings as a source of information were the first, second, and third most frequent responses among doctors (Figure 6B).

### 3.3 Analysis of criteria that may influence the selection of a drug for the treatment of allergic rhinitis

#### 3.3.1 Gender as a criterion for drug selection in the treatment of allergic rhinitis

Using contingency tables, we analyzed whether the gender of doctors may affect the selection of a drug for the treatment of allergic rhinitis. As shown in Supplementary Table S1, gender was significantly related to the average number of patients seen per week; 33% of female participants and 30.3% of male participants had 51–100 patients weekly (X-squared = 11.739, df = 5, *p*-value = 0.03854, Cramer’s V = 0.1978159).

Intranasal glucocorticoids and oral antihistamines were the most frequently used treatment in patients aged 5-18, according to 81.7% and 80.4% of female respondents and 65.8% and 69.7% of male respondents (X-squared = 17.81, df = 9, *p*-value = 0.03745, Cramer’s V = 0.1700349) (Supplementary Table S4).

**FIGURE 7:**
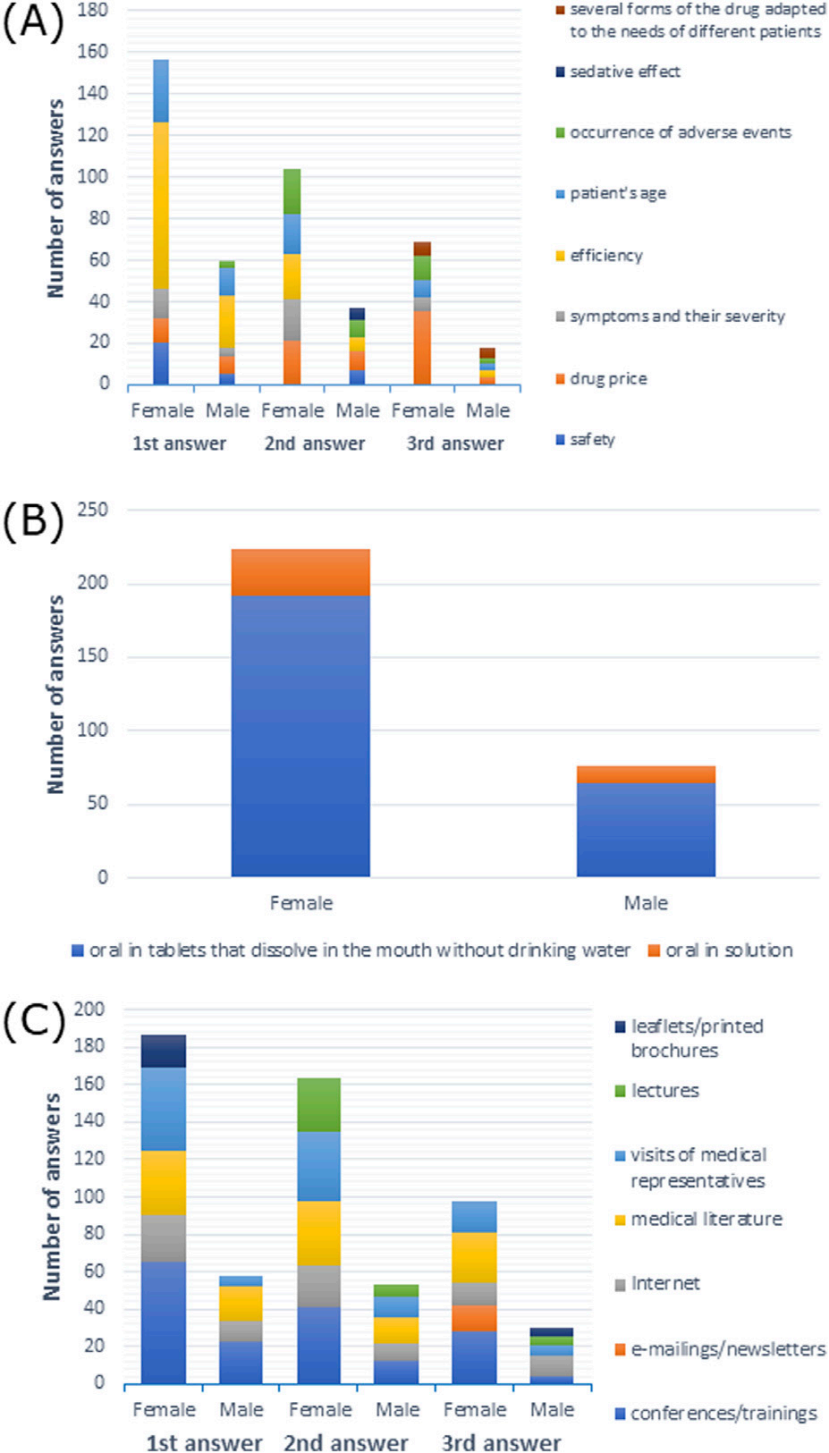
**(A)** Most commonly indicated of the three most important factors affecting drug choice; **(B)** route of administration preferred by patients; **(C)** most frequently indicated of the three most important sources of information depending on gender.

Efficiency of a drug was the most frequently reported response among 48.7% of female and male respondents (*p* > 0.05) (Supplementary Table S6). Efficiency was also the most frequent first response for both female and male participants, although efficiency and occurrence of adverse events were the most frequent second response among female respondents. Drug price was the most frequent second response among male respondents and the most frequent third response among female participants (Figure 7A).

When it came to drug administration routes preferred by patients, 91.1% of female respondents and 89.5% of male respondents preferred the oral route (*p* > 0.05) (Supplementary Table S7). As shown in Figure 7B, tablets that dissolve in the mouth without drinking water were the most frequently reported option among both female and male participants.

As shown in Supplementary Table S9, 31–50 PLN (∼€7–€11) was the price that the patients were willing to pay for the treatment of moderate forms of AR, which was reported by 35.7% of female participants and 34.2% of male participants (X-squared = 14.182, df = 6, *p*-value = 0.02767, Cramer’s V = 0.2174235).

Among female participants, 46.0% preferred visits from medical representatives as a source of medical information, while 54.7% of male participants opted for information from conferences/training (*p* > 0.05) (Supplementary Table S13). As shown in Figure 7C, conferences/training as a source of information was the most common first, second, and third response for female participants, and it was the most common first response among male participants. The second and third most common responses among male participants were medical literature and the internet, respectively.

Unfortunately, the remaining results were statistically insignificant (Supplementary Tables S2, S3, S5, S8, S10–S12).

#### 3.3.2 Main workplace as a criterion for drug selection in the treatment of allergic rhinitis

Next, we analyzed whether the main workplace of the participants may impact the selection of a drug for the treatment of allergic rhinitis. Of the respondents employed in private primary care clinics, 33.3% had between 21–50 and 101–150 patients weekly; 38.4% employed in private specialist clinics had 51–100 patients weekly; 35% from private practices had ≤20 patients per week; and 60% from public primary care clinics, 29.4% from public specialist clinics, and 42.3% from other places had 51–100 patients (X-squared = 66.63, df = 25, *p*-value = 1.203e-05, Cramer’s V = 0.2111127) (Supplementary Table S14). Fifty percent of respondents working in private primary care clinics, 38.4% in private specialist clinics, and 80% from public primary care clinics had, on average, 21–50 patients with AR per week; 57.5% of respondents from private practices and 42.3% from other places had ≤20 patients with AR per week; and 36.4% from public specialist clinics had 51–100 patients with AR weekly (X-squared = 37.654, df = 15, *p*-value = 0.001015, Cramer’s V = 0.2048836) (Supplementary Table S15).

Intranasal glucocorticoids and oral antihistamines were the most frequently prescribed treatments in patients over 18 years of age: 83.3% and 100% from private primary care clinics, 90.4% and 89% from private specialist clinics, 92.5% and 80% from private practices, 60% from public primary care clinics, 81.1% and 77.6% from public specialist clinics, and 77.8% and 81.5% from other places (X-squared = 114.81, df = 50, *p*-value = 5.222e-07, Cramer’s V = 0.1910661). For patients between 5 and 18 years of age: 66.7% and 83.3% from private primary care clinics, 86.3% and 91.8% from private specialist clinics, 77.5% and 70% from private practices, 60% and 100% from public primary care clinics, 74.1% and 69.9% from public specialist clinics, and 81.5% and 85.2% from other places (X-squared = 95.492, df = 45, *p*-value = 1.707e-05, Cramer’s V = 0.1760791), as shown in Supplementary Tables S16–S17).

**FIGURE 8:**
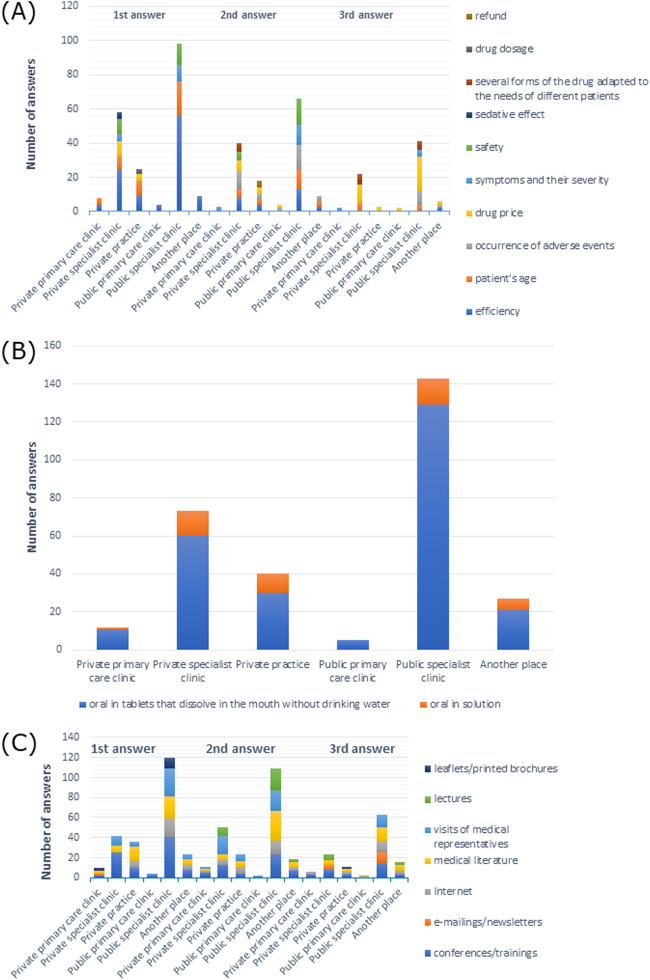
**(A)** Most commonly indicated of the three most important factors in choosing a drug; **(B)** route of administration preferred by patients; **(C)** most frequently indicated of the three most important sources of information depending on the main workplace.

Symptoms and their severity were the most frequently chosen responses by participants from private primary care clinics (50%), but in the case of other workplaces, efficiency was the prevailing response: 54.8% from private specialist clinics, 45% from private practices, 80% from public primary care clinics, 47.6% from public specialist clinics, and 48.1% from other places (X-squared = 122.81, df = 90, *p*-value = 0.0123, Cramer’s V = 0.1787216), as shown in Supplementary Table S19. Efficiency was also the most common first response across all types of workplaces (Figure 8A).

When it came to drug administration routes preferred by patients, 100% of doctors from private primary care clinics, 89% from private specialist clinics, 85% from private practices, 100% from public primary care clinics, 91.6% from public specialist clinics, and 92.6% from other places preferred the oral form (*p* > 0.05) (Supplementary Table S20). In particular, tablets that dissolve in the mouth without drinking water were the most frequently reported form of medication across all types of workplaces, as shown in Figure 8B.

Among doctors from private primary care clinics, 58.3% preferred information at conferences/training as a source of information, 52.8% from private specialist clinics 45.5% from public specialist clinics preferred visits from medical representatives, 42.5% from private practices preferred the Internet, and 80% from public primary care clinics and 59.3% from other places preferred literature (Supplementary Table S26). As shown in Figure 8C, the information provided at conferences/training was the most frequently reported first response among participants from private specialist clinics, public specialist clinics, and other places, while it was the second most common response from private primary care clinics and other places, and the third answer from private specialist clinics and public specialist clinics, and most often reported by doctors from private practices. Medical literature was most commonly given as the first response among participants from private practices, the second response from doctors working in public specialist clinics, and the third response from physicians working in other places. Visits by medical representatives were the most common second response in private specialist clinics.

Unfortunately, other results were statistically insignificant (Supplementary Tables S18, S21–S25).

#### 3.3.3 Size of the town of the main workplace as a criterion for drug selection in the treatment of allergic rhinitis

Next, using contingency tables, we analyzed whether the population of the town where a doctor’s main workplace was located may affect a drug choice for the treatment of AR. Of the doctors from cities with <50 thousand inhabitants, 24.1% had 101–150 patients weekly, while 36.4% of doctors from cities of 50–100 thousand inhabitants and 33.9% of doctors from cities >100 thousand inhabitants had 51–100 patients/week (X-squared = 21.423, df = 10, *p*-value = 0.01833, Cramer’s V = 0.1889586) (Supplementary Table S27).

Intranasal glucocorticoids and oral antihistamines were the most frequently used treatment in patients over 18 years of age by 81.5% and 77.8% of doctors from cities with <50 thousand inhabitants, 78.8% and 75.8% from cities with 50–100 thousand inhabitants, and 87.2% and 85.0% from cities with >100 thousand inhabitants (X-squared = 45.512, df = 20, *p*-value = 0.00094, Cramer’s V = 0.1902054). In the case of patients up to 5 years of age, intranasal glucocorticoids and oral antihistamines were prescribed by 48.1% and 74.1% of doctors from cities with <50 thousand inhabitants, 39.4% and 51.5% from cities with 50–100 thousand inhabitants, and 43.9% and 63.9% from cities with >100 thousand inhabitants, respectively (X-squared = 27.811, df = 14, *p*-value = 0.01507, Cramer’s V = 0.1742441), as shown in Supplementary Tables S29, S31.

**FIGURE 9:**
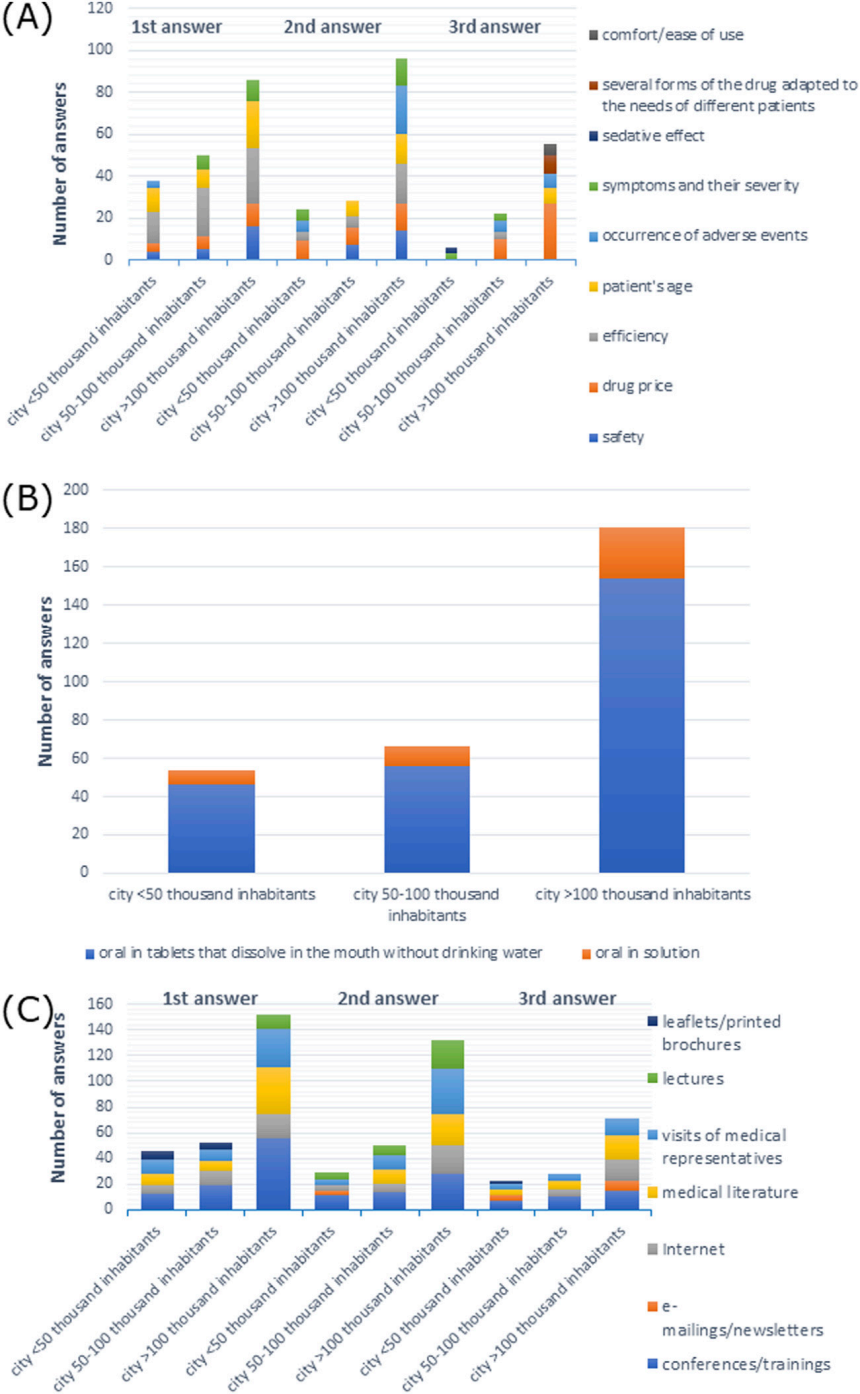
**(A)** Most commonly indicated of the three most important factors affecting the choice of a drug; **(B)** route of administration preferred by patients; **(C)** most frequently indicated of the three most important sources of information depending on the size of the town of the main workplace.

The efficiency of drugs was the most frequently named answer: 42.6% of doctors from cities with <50 thousand inhabitants, 53% from cities with 50–100 thousand inhabitants, and 48.9% from cities with >100 thousand inhabitants, respectively (*p* > 0.05) (Supplementary Table S32). Efficiency was also the most common first response among all participants. Drug price was the most commonly reported second response among participants from cities with <50 and 50–100 thousand inhabitants, and the most frequent third response among participants from cities with 50–100 and >100 thousand inhabitants, while adverse events were the most frequent second response among participants from cities with >100 thousand inhabitants (Figure 9A).

When it came to drug administration routes preferred by patients, 96.3% of participants from cities with <50 thousand inhabitants, 84.8% from cities with 50–100 thousand inhabitants, and 91.1% from cities with >100 thousand inhabitants preferred the oral route of administration (X-squared = 15.754, df = 8, *p*-value = 0.04603, Cramer’s V = 0.1504511) (Supplementary Table S33). As shown in Figure 9B, tablets that dissolve in the mouth without drinking water were the most common response among all participants.

The majority of the participants preferred visits by medical representatives and information at conferences/training as a source of information: 38.9% and 38.9% of participants from cities with <50 thousand inhabitants, 43.9% and 48.5% from cities with 50–100 thousand inhabitants, and 44.1% and 48.0% from cities with >100 thousand inhabitants, respectively, (*p* > 0.05) which is shown in Supplementary Table S39. As shown in Figure 9C, conferences/training as a source of information was the most frequent first response among all participants, and it was the second and the third response among doctors from cities with <50 and 50–100 thousand inhabitants, respectively. Visits from medical representatives were the most frequent second response, whereas medical literature was the most frequent third response among participants from cities with >100 thousand inhabitants.

Unfortunately, other results were statistically insignificant (Supplementary Tables S30, S34–S38).

#### 3.3.4 Voivodeship of the main workplace as a criterion for drug selection in the treatment of allergic rhinitis

Next, we analyzed whether the voivodeship in which the doctor’s main workplace was located may affect the selection of a drug for the treatment of AR; 27.8% of doctors from Dolnośląskie Voivodeship, 40% from Kujawsko-Pomorskie Voivodeship, 44.4% from Lubelskie Voivodeship, and 42.9% from Wielkopolskie Voivodeship had 21–50 patients weekly; 47.1% from Łódzkie Voivodeship, 45% from Małopolskie Voivodeship, 52.3% from Mazowieckie Voivodeship, and 25% from Podlaskie Voivodeship had 51–100 patients weekly; 100% from Lubuskie Voivodeship, 70% from Podkarpackie Voivodeship, 55.6% from Świętokrzyskie Voivodeship, and 50% from Zachodniopomorskie Voivodeship had 101–150 patients weekly; 31.8% from Śląskie Voivodeship had 151–200 patients weekly; 33.3% from Pomorskie Voivodeship had ≥201 patients weekly; 33.3% from Warmińsko-Mazurskie Voivodeship had 21–50 and 101–150 patients weekly; and 30% from Opolskie Voivodeship had 51–100 and 101–150 patients weekly (X-squared = 161.69, df = 75, *p*-value = 2.621e-08, Cramer’s V = 0.3283238), as shown in Supplementary Table S40). Supplementary Table S41 shows that 55.6% of doctors from Dolnośląskie Voivodeship and 60% from Kujawsko-Pomorskie Voivodeship had ≤20 patients per week with AR; 61.1% from Lubelskie Voivodeship, 44.1% from Łódzkie Voivodeship, 45% from Małopolskie Voivodeship, 50% from Opolskie Voivodeship, and 35.7% from Wielkopolskie Voivodeship had 21–50 patients per week with AR; 75% from Lubuskie Voivodeship, 36.4% from Mazowieckie Voivodeship, 45.4% from Podlaskie Voivodeship, 40.9% from Śląskie Voivodeship, 50% from Świętokrzyskie Voivodeship, 50% from Warmińsko-Mazurskie Voivodeship, and 66.7% from Zachodniopomorskie Voivodeship had 51–100 patients weekly with AR; 40% from Podkarpackie Voivodeship had ≤20 and 51–100 patients weekly with AR; and 33.3% from Pomorskie Voivodeship had ≤20 and 21–50 patients per week with AR (X-squared = 75.59, df = 45, *p*-value = 0.002893, Cramer’s V = 0.289809).

Intranasal glucocorticoids and oral antihistamines were the most frequently used treatments in patients over 18 years of age: 100% and 88.9% of doctors from Dolnośląskie Voivodeship, 100% and 80% from Kujawsko-Pomorskie Voivodeship, 77.8% and 77.8% from Lubelskie Voivodeship, 50% and 50% from Lubuskie Voivodeship, 76.5% and 82.4% from Łódzkie Voivodeship, 90% and 90% from Małopolskie Voivodeship, 86.4% and 81.8% from Mazowieckie Voivodeship, 100% and 100% from Opolskie Voivodeship, 90% and 90% from Podkarpackie Voivodeship, 78.6% and 78.6% from Podlaskie Voivodeship, 77.8% and 77.8% from Pomorskie Voivodeship, 90.9% and 90.9% from Śląskie Voivodeship, 77.8% and 77.8% from Świętokrzyskie Voivodeship, 100% and 71.4% from Wielkopolskie Voivodeship, and 66.7% and 66.7% from Zachodniopomorskie Voivodeship, respectively, while 33.3% of respondents from Warmińsko-Mazurskie Voivodeship answered oral antihistamines, topical antihistamines, intranasal glucocorticoids, and systemic glucocorticoids, and 33.3% did not have such patients (X-squared = 259.09, df = 150, *p*-value = 7.893e-08, Cramer’s V = 0.2029546). Between 5 and 18 years of age, 88.9% and 77.8% of doctors from Dolnośląskie Voivodeship, 100% and 80% from Kujawsko-Pomorskie Voivodeship, 88.9% and 83.3% from Lubelskie Voivodeship, 64.7% and 64.7% from Łódzkie Voivodeship, 95% and 95% from Małopolskie Voivodeship, 75% and 68.2% from Mazowieckie Voivodeship, 100% and 100% from Opolskie Voivodeship, 100% and 100% from Podkarpackie Voivodeship, 50% and 50% from Podlaskie Voivodeship, 83.3% and 83.3% from Pomorskie Voivodeship, 72.7% and 100% from Śląskie Voivodeship, 83.3% and 72.2% from Świętokrzyskie Voivodeship, 85.7% and 57.1% from Wielkopolskie Voivodeship, and 66.7% and 100% from Zachodniopomorskie Voivodeship, respectively, while 67.7% from Warmińsko-Mazurskie Voivodeship answered oral antihistamines and topical antihistamines, and 100% from Lubuskie Voivodeship responded oral antihistamines (X-squared = 222.64, df = 135, *p*-value = 3.057e-06, Cramer’s V = 0.2003982). For patients up to 5 years of age: 33.3% and 88.9% of doctors from Dolnośląskie Voivodeship, 60% and 60% from Kujawsko-Pomorskie Voivodeship, 77.8% and 88.9% from Lubelskie Voivodeship, 35.3% and 44.1% from Łódzkie Voivodeship, 36.4% and 61.4% from Mazowieckie Voivodeship, 80% and 60% from Opolskie Voivodeship, 40% and 80% from Podkarpackie Voivodeship, 94.4% and 83.3% from Pomorskie Voivodeship, 45.5% and 81.8% from Śląskie Voivodeship, and 55.6% and 66.7% from Świętokrzyskie Voivodeship, respectively, but 50% from Małopolskie Voivodeship, 57.1% from Wielkopolskie Voivodeship, and 100% from Zachodniopomorskie Voivodeship responded oral antihistamines, 42.9% from Podlaskie Voivodeship responded intranasal glucocorticoids, and 42.9% did not have such patients (X-squared = 191.64, df = 105, *p*-value = 5.101e-07, Cramer’s V = 0.2444914), as shown in Supplementary Tables S42–S44).

**FIGURE 10:**
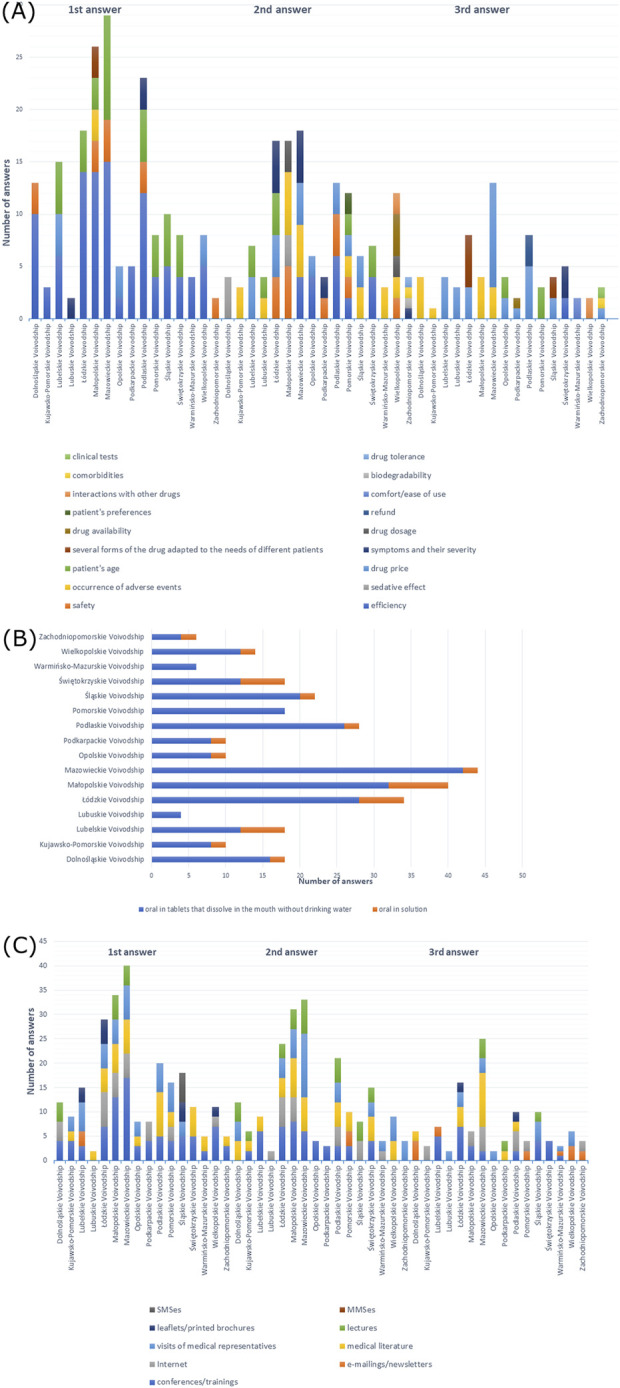
**(A)** Most commonly indicated of the three most important factors in choosing a drug; **(B)** route of administration preferred by patients; **(C)** most frequently indicated of the three most important sources of information depending on the voivodeship of the main workplace.

Efficiency of a drug was the most frequently reported among doctors: 66.7% from Dolnośląskie Voivodeship, 50% from Kujawsko-Pomorskie Voivodeship, 47.1% from Łódzkie Voivodeship, 35% from Małopolskie Voivodeship, 63.9% from Mazowieckie Voivodeship, 50% from Podkarpackie Voivodeship, 60.7% from Podlaskie Voivodeship, 50% from Świętokrzyskie Voivodeship, 66.7% from Warmińsko-Mazurskie Voivodeship, and 33.3% from Zachodniopomorskie Voivodeship, but 50% from Lubelskie Voivodeship, 55.6% from Pomorskie Voivodeship, 40.9% from Śląskie Voivodeship responded patient’s age, 70% from Opolskie Voivodeship and 57.1% from Wielkopolskie Voivodeship answered drug price, and 50% from Lubuskie responded efficiency, patient’s age, drug price, refund, as well as symptoms and their severity (X-squared = 440.75, df = 270, *p*-value = 2.378e-10, Cramer’s V = 0.1954731) (Supplementary Table S45). Efficiency was also the most frequent first response in the majority of voivodeships (Figure 10A).

When it came to drug administration routes preferred by patients, the majority of doctors from all voivodeships responded with the oral form (*p* > 0.05) (Supplementary Table S46). As shown in Figure 10B, oral tablets that dissolve in the mouth without drinking water were most frequently reported among all participants.

As shown in Supplementary Table S47, 16–30 PLN (∼€3–€7) was the price that the patients were willing to pay for mild forms of the disease, which was reported by 41.2% of doctors from Łódzkie Voivodeship, 35.7% from Podlaskie Voivodeship, 44.4% from Pomorskie Voivodeship, and 66.6% from Zachodniopomorskie Voivodeship; 31–50 PLN (∼€7–€11) was the price answered by 66.7% of participants from Dolnośląskie Voivodeship, 35% from Małopolskie Voivodeship, 36.4% from Mazowieckie Voivodeship, 40% from Opolskie Voivodeship, 40% from Podkarpackie Voivodeship, and 36.4% from Śląskie Voivodeship. However, 30% of doctors from Kujawsko-Pomorskie Voivodeship and 33.3% from Warmińsko-Mazurskie Voivodeship responded 31–50 (∼€7–€11) and ≥51 PLN (∼€11) and 30% of participants also had difficulty answering; 38.9% from Lubelskie Voivodeship responded 31–50 PLN (∼€7–€11), and a similar percentage of participants had difficulty answering; 50% from Lubuskie Voivodeship and 28.6% from Wielkopolskie Voivodeship answered 16–30 (∼€3–€7) and 31–50 PLN (∼€7–€11), respectively; 38.9% from Świętokrzyskie Voivodeship had difficulty answering (X-squared = 120.37, df = 90, *p*-value = 0.01794, Cramer’s V = 0.2585959). In the moderate form of AR, 75% from Lubuskie Voivodeship, 41.2% from Łódzkie Voivodeship, 32.5% from Małopolskie Voivodeship, 43.2% from Mazowieckie Voivodeship, 35.7% from Podlaskie Voivodeship, 36.4% from Śląskie Voivodeship, 42.9% from Wielkopolskie Voivodeship, and 83.3% from Zachodniopomorskie Voivodeship answered 31–50 PLN (∼€7–€11); 55.6% of doctors from Dolnośląskie Voivodeship, 40% from Kujawsko-Pomorskie Voivodeship, 50% from Opolskie Voivodeship, 60% from Podkarpackie Voivodeship, and 50% from Pomorskie Voivodeship responded 51–100 PLN (∼€11–€22); 33.3% from Warmińsko-Mazurskie Voivodeship responded 31–50 (∼€7–€11) and 51–100 PLN (∼€11–€22), and a similar percentage of participants had difficulty answering; 50% from Lubelskie Voivodeship and 38.9% from Świętokrzyskie Voivodeship had difficulty answering (X-squared = 127.12, df = 90, *p*-value = 0.006105, Cramer’s V = 0.2657475) (Supplementary Table S48). In the severe form, the majority of participants responded 51–100 PLN (∼€11–€22) (38.9% from Dolnośląskie Voivodeship, 60% from Kujawsko-Pomorskie Voivodeship, 50% from Lubelskie Voivodeship, 75% from Lubuskie Voivodeship, 47.1% from Łódzkie Voivodeship, 56.8% from Mazowieckie Voivodeship, 40% from Opolskie Voivodeship, 60% from Podkarpackie Voivodeship, 44.4% from Pomorskie Voivodeship, 36.4% from Śląskie Voivodeship, and 83.3% from Zachodniopomorskie Voivodeship), while 27.5% from Małopolskie Voivodeship and 35.7% from Podlaskie Voivodeship answered 51–100 (∼€11–€22) and 101–200 PLN (∼€22–€46), respectively; 35.7% from Wielkopolskie Voivodeship responded ≤50 (∼€11) and 51–100 PLN (∼€11–€22); 33.3% from Warmińsko-Mazurskie Voivodeship responded 51–100 PLN (∼€11–€22) and had difficulty answering; 38.9% from Świętokrzyskie Voivodeship had difficulty answering (X-squared = 117.87, df = 90, *p*-value = 0.02597, Cramer’s V = 0.2559015) (Supplementary Table S49); 72.2% of doctors from Lubelskie Voivodeship, 52.9% from Łódzkie Voivodeship, 50% from Małopolskie Voivodeship, 60% from Opolskie Voivodeship, 63.6% from Śląskie Voivodeship, and 47.1% from Świętokrzyskie Voivodeship responded that patients choose a more expensive drug for 2 months of therapy; 40.9% from Mazowieckie Voivodeship and 66.7% from Pomorskie Voivodeship responded that the patients choose a cheaper drug for 1 month; while 60% from Podkarpackie Voivodeship and 66.7% from Warmińsko-Mazurskie Voivodeship had difficulty answering. However, 44.4% from Dolnośląskie Voivodeship, 40% from Kujawsko-Pomorskie Voivodeship, and 42.9% from Podlaskie Voivodeship gave both answers; 50% from Lubuskie Voivodeship and 42.9% from Wielkopolskie Voivodeship thought that patients would choose a more expensive drug that would be enough for 2 months of therapy and, simultaneously, had difficulty answering; and 33.3% from Zachodniopomorskie Voivodeship responded with all answers (X-squared = 54.124, df = 30, *p*-value = 0.004453, Cramer’s V = 0.300847), as shown in Supplementary Table S50. However, there was no statistically significant dependence between the voivodeship of the main workplace and the cost that the patient was willing to spend monthly for all medications used (Supplementary Table S51).

In total, 50% of participants from Małopolskie Voivodeship, 61.1% from Świętokrzyskie Voivodeship, and 64.3% from Wielkopolskie Voivodeship preferred information provided at conferences/trainings as the source of information, while 100% from Lubuskie Voivodeship, 54.5% from Mazowieckie Voivodeship, 39.3% from Podlaskie Voivodeship, and 83.3% from Zachodniopomorskie Voivodeship preferred visits of medical representatives, while 40.9% from Śląskie Voivodeship, 70% from Opolskie Voivodeship, and 45.5% from Łódzkie Voivodeship preferred both options; 70% of participants from Kujawsko-Pomorskie Voivodeship preferred literature, and 66.7% from Warmińsko-Mazurskie Voivodeship preferred information at conferences/training and literature; 44.4% from Dolnośląskie Voivodeship and 50% from Lubelskie Voivodeship preferred e-mailings/newsletters; and 80% from Podkarpackie Voivodeship and 44.4% from Pomorskie Voivodeship preferred Internet (X-squared = 285.34, df = 180, *p*-value = 9.326e-07, Cramer’s V = 0.1821084), which is shown in Supplementary Table S39. Figure 10C shows the distribution of the most frequent answers to the last question.

#### 3.3.5 Work experience as a criterion for drug selection in the treatment of allergic rhinitis

Next, using contingency tables, we analyzed whether the work experience (after board certification) of doctors may influence the choice of a drug for the treatment of AR; 39% of doctors with work experience ≤10 years, 39.2% of doctors with work experience ≥31 years, and 50% of doctors with undefined work experience had ≤20 patients with AR weekly, whereas 38.6% of doctors with work experience 11–20 years, 39.8% of doctors with work experience 21–30 years, and 50% of doctors with undefined work experience had 21–50 patients with AR per week (X-squared = 23.437, df = 12, *p*-value = 0.02424, Cramer’s V = 0.1613725) (Supplementary Table S54).

Intranasal glucocorticoids and oral antihistamines were the most frequently used treatments by doctors in patients aged 5 -18 years: 87.8% and 78% of doctors with work experience ≤10 years, 88.6% and 85.7% of doctors with work experience 11–20 years, 74.3% and 73.5% of doctors with work experience 21–30 years, 67.6% and 77% of doctors with work experience ≥31 years, and 50% and 50% of doctors with undefined work experience, respectively, (X-squared = 56.325, df = 36, *p*-value = 0.01669, Cramer’s V = 0.1511925) and in patients up to 5 years of age, which was the response of 46.3% and 70.7% of doctors with work experience ≤10 years, 50% and 72.9% of doctors with work experience 11–20 years, 46% and 68.1% of doctors with 21–30 years, and 33.8% and 41.9% of doctors with work experience ≥31 years (40.5% of doctors did not have such patients), respectively, but 50% of doctors with undefined work experience preferred oral antihistamines and cromones and also did not have such patients (X-squared = 55.546, df = 28, *p*-value = 0.001461, Cramer’s V = 0.1741261), as shown in Supplementary Tables S56, S57.

**FIGURE 11:**
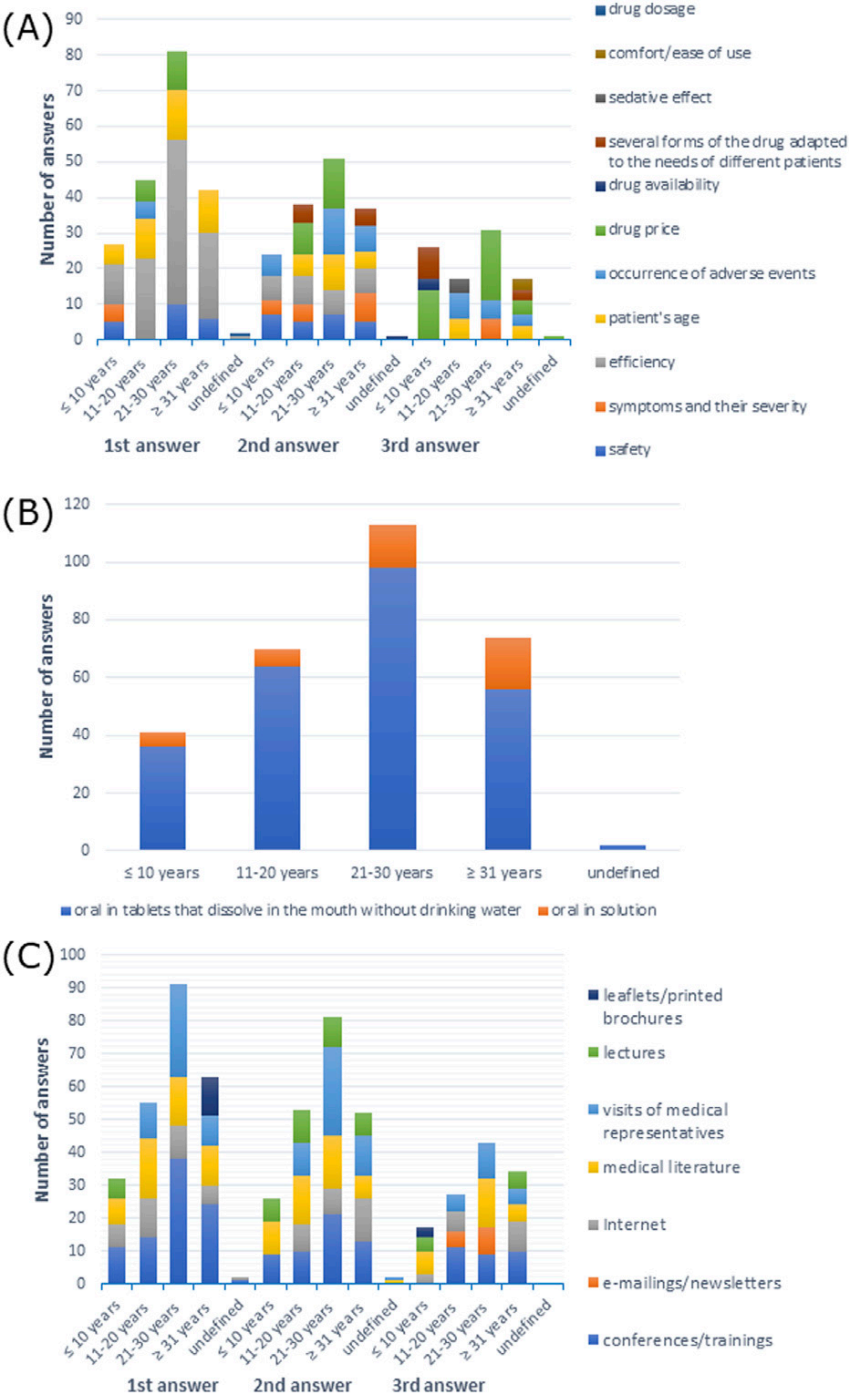
**(A)** Most commonly indicated of the three most important factors in choosing a drug; **(B)** route of administration preferred by patients; **(C)** most frequently indicated of the three most important sources of information depending on work experience.

Efficiency of a drug was the most frequently reported answer, but not the only one, although it was the most common response among doctors with undefined work experience (*p* > 0.05) (Supplementary Table S58). Efficiency was also the most common first response among all participants. The second response was drug price among participants with 11–20 and 21–30 years of work experience, symptoms and their severity among participants with work experience ≥31 years, safety and efficiency among participants with work experience ≤10 years, and drug availability among participants with undefined work experience. The third response was drug price among participants with work experience ≤10 and 21–30 years and participants with undefined work experience, occurrence of adverse events among participants with work experience 11–20 years, and patient’s age and drug price among participants with work experience ≥31 years (Figure 11A).

The oral route was the most preferred drug administration route by patients (*p* > 0.05) (Supplementary Table S59). As shown in Figure 11B, oral tablets that dissolve in the mouth without drinking water were the most frequently reported answer among all participants.

As shown in Supplementary Table S60, 16–30 PLN (∼€3–€7) was the price that patients were willing to pay for treating the mild form of the disease according to the response of 37.1% of doctors with work experience 11–20 years, 51.2% of doctors with work experience ≤10 years, 37.2% of doctors with work experience 21–30 years; 50% of doctors with undefined work experience (50% also indicated other answer) answered 31–50 PLN (∼€7–€11), but 31.1% of doctors with work experience ≥31 years had difficulty answering (X-squared = 51.837, df = 24, *p*-value = 0.0008217, Cramer’s V = 0.2078391). In treatment of the moderate form of AR, 35.7% of doctors with work experience 11–20 years and 42.5% of doctors with work experience 21–30 years responded 31–50 PLN (∼€7–€11), but 46.3% of doctors with work experience ≤10 years and 50% of doctors with undefined work experience (50% also gave another answer) answered 51–100 PLN (∼€11–€22), and 31.1% of doctors with work experience ≥31 years had difficulty answering (X-squared = 68.153, df = 24, *p*-value = 4.149e-06, Cramer’s V = 0.238315) (Supplementary Table S61). In the treatment of the severe form, the majority of the participants responded 51–100 PLN (∼€11–€22), and 50% of doctors with undefined work experience also responded with other answers (X-squared = 72.3, df = 24, *p*-value = 9.752e-07, Cramer’s V = 0.2454585) (Supplementary Table S62). When it came to the cost that the patients were willing to spend monthly on all medications used, the majority of doctors with work experience 21–30 and ≥31 years had difficulty answering, but 26.8% of doctors with work experience ≤10 years responded 151–200 PLN (∼€35–€46), and 24.3% of doctors with work experience 11–20 years answered ≤100 (∼€22) and 101–150 PLN (∼€22–€35). However, 50% of doctors with undefined work experience had difficulty answering and responded with other answers (X-squared = 66.304, df = 28, *p*-value = 6.01e-05, Cramer’s V = 0.23506), as shown in Supplementary Table S64. However, there was no statistically significant dependence between the voivodeship of the doctor’s main workplace and the patient’s choice between a cheaper drug for 1 month and a more expensive drug for 2 months of therapy (Supplementary Table S63).

The majority of the participants preferred information provided at conferences/training: 43.4% of doctors with work experience of 21–30 years, 58.1% of doctors with work experience ≥31 years, and 100% of doctors with undefined work experience; 43.9% of doctors with work experience ≤10 years preferred literature, and 44.9% of doctors with work experience 11–20 years preferred Internet (X-squared = 89.892, df = 48, *p*-value = 0.0002363, Cramer’s V = 0.1770397), which is shown in Supplementary Table S65. As shown in Figure 11C, conferences/training as a source of information was the first response among all participants, except for doctors with work experience of 11–20 years, who preferred medical literature.

## 4 Discussion

In our survey study, we analyzed the criteria that influence doctors’ decision-making regarding the treatment of allergic rhinitis (AR). We analyzed five different criteria, which were gender, main workplace, size of the town where the main workplace is located, voivodeship of the main workplace, and work experience. In general, participating doctors had 51–100 patients weekly, including 21–50 patients with AR. The majority of the participants preferred information from conferences/training and visits from medical representatives as sources of information. However, these results differed between the analyzed groups.

Modern medicine relies on the standards provided by evidence-based medicine (EBM), which describes step by step how to choose and evaluate relevant medical data and highlights the importance of communication between a physician and a patient as a tool for understanding the patient’s needs and preferences (Tenny and Varacallo, 2023). According to the International Consensus Statement on Allergy and Rhinology: Allergic Rhinitis 2023 (ICAR-Allergic Rhinitis 2023) (Wise et al., 2023), in which the authors summarized evidence-based recommendations regarding allergic rhinitis, oral H1 antihistamines, and intranasal corticosteroid spray were strongly recommended based on well-designed, randomized controlled trials. In 2023, oral corticosteroids and oral decongestants were also strongly recommended in comparison to 2018 but based on randomized, controlled trials with some limitations and overwhelming, consistent evidence from observational studies. Our analyses showed that doctors most often prescribe intranasal glucocorticoids and oral antihistamines to treat allergic rhinitis in patients of all age groups. Similarly, oral antihistamines and intranasal corticosteroids were the most commonly prescribed medications among AR patients in the study conducted in Italy. However, merely 33.5% of patients were satisfied with the proposed treatments (Ciprandi et al., 2011).

The concept of shared decision-making (SDM) has been proposed by multiple medical organizations and seems to be crucial in cases of chronic diseases that require complex treatment. In the case of AR, SDM is applicable due to the chronic nature of the disease and the impact that the disease has on a patient’s daily life. In this case, communication between the physician and the patient is especially important as different therapeutical approaches are available, e.g., whether the patient prefers a sublingual tablet or a subcutaneous injection (Blaiss et al., 2019). According to our analysis, in the opinion of allergologists, patients generally preferred sublingual tablets. Of note, sublingual tablets are generally widely accepted by patients, as local therapies with a low margin of side effects and high efficiency are preferred (Titulaer et al., 2018). Moreover, sublingual administration decreases the indirect costs of treatment when compared to subcutaneous immunotherapy for AR, as due to its formulation, the drug does not require it to be administered by trained medical personnel (Cox et al., 2020). Furthermore, communication and trust between a doctor and a patient are essential in AR management. Some patients suffering from AR often experience difficulties in the diagnosis, which in turn, results in frustration and mistrust of medical care professionals. Despite the fact that patients may comply with doctor recommendations in the long term, they fail to return to the office for follow-up visits as they often manage the disease according to their health beliefs (Cvetkovski et al., 2019). A multicenter study conducted in Hungary and Spain showed that most patients with uncontrolled symptoms of AR, who were previously treated with standard treatment (e.g., intranasal glucocorticoids or a combination of intranasal glucocorticoids and antihistamine drugs), had their treatment modified according to the guidelines; while in the case of 27% of patients in Hungary and 40% in Spain, doctors failed to adjust the treatment. Although the infectivity of treatment might have partially resulted from a lack of compliance with the doctor’s recommendations, in some cases, patients did not receive proper therapy, which highlights an urgent need for additional training for doctors in the treatment of allergic rhinitis (Gálffy et al., 2021). Additionally, we analyzed the factors that affect the decision-making related to the choice of drugs for AR treatment: the majority of doctors picked the efficiency of the drug. Statistical analysis showed a significant relationship between the factors and the main workplace and the voivodeship of the main workplace. Among the factors, the most popular responses, except efficiency, were symptoms and their severity, drug price, and patients’ age. Similarly, efficiency was most often chosen first response in general, but it also depended on the analyzed criteria.

In addition to the issues related to trust and communication, there is the economic factor; the direct and indirect costs of AR management are a serious burden worldwide. A study conducted on Swedish AR patients showed that the average cost of AR treatment is €961.1 per patient annually, which on the country scale amounts approximately to €1.3 billion (Cardell et al., 2016). In Beijing (China), the cost of the yearly treatment equals €195.6 per patient, which corresponds to €440.9 million at the city level (Li et al., 2022). In 2013, in the United States (US), the estimated cost of rhinitis management equaled $3.3 billion. According to a study by Roland et al. (2010), the annual cost of AR medications is $109 per patient, which equals to $1.3 for the whole of the US; whereas the total cost of treatment is expected to reach >$4.6 billion. In our study, respondents claimed that patients are willing to pay 31–50 PLN (∼€7- €11) monthly for the treatment of allergic rhinitis in mild and moderate forms, while they were willing to pay 51–100 PLN (∼€11–€22) for treating the severe form. The aforementioned costs are relatively high, considering that, according to the Statistics Poland, the median monthly salary was 4702.66 (€1055.56) in October 2020; therefore, the cost of AR treatment could be a substantial burden, especially for less affluent citizens (GUS, 2023). Moreover, we found significant relationships between the price that the patients were willing to pay for moderate forms and gender, voivodeship of the main workplace, and work experience; the price that the patients were willing to pay for mild and severe forms and voivodeship of the main workplace and work experience; the choice of a cheaper drug for 1 month or a more expensive drug for 2 months of therapy and the voivodeship of the main workplace; and the price that is the patients were willing to spend per month for all the medications used and work experience. Interestingly, the cost of AR management could be reduced if the patients received proper treatment in compliance with guidelines and looked for professional medical care instead of self-medicating (Avdeeva et al., 2020).

Our survey study has its strengths and weak points. On the one hand, the study was conducted on a large number of doctors working in various centers throughout Poland using a self-administered questionnaire. This allowed us to paint a relatively clear picture of the differences in the therapy choice predictors among doctors practicing across the country. Therefore, we can be sure that the results of our study are not subject to selectivity bias. On the other hand, the study used CATI, which, in addition to being accurate in collecting data, saving time, and being less error-prone than paper surveys, can cause problems in the case of open-ended questions. 

Despite this, our study confirmed that the management of AR should be focused on the patient. One of the most important factors in choosing a drug is its effectiveness. Moreover, an important factor in the effective treatment of AR is the financial issue, and as our analysis shows, AR treatment costs can be a significant burden, especially for the less affluent citizens in Poland.

## Data Availability

The original contributions presented in the study are included in the article/Supplementary Material; further inquiries can be directed to the corresponding author.
