# Supplementary material for: Analysis of criteria for choosing drug treatment strategies in allergic rhinitis
Source: Front Pharmacol. 2024 Oct 1;15:1340554. doi: 10.3389/fphar.2024.1340554 (PMC11473967; doi:10.3389/fphar.2024.1340554)
Supplement: Supplementary file 1 [file DataSheet1.docx]

**Study: Analysis of the costs of treating patients with allergic rhinitis.**

Hello, my name is............ I represent the QAH research agency. We conduct research with allergists on allergic rhinitis (AR). Therefore, I would like to ask you a few questions. The interview will be short and completely anonymous, and your statements will be analyzed only in the form of collective statistical studies.

**DOCTOR'S METRICS**

1. **Gender:**

□ female □ male

1. **Main workplace:**

- Public specialist clinic
- Private specialist clinic
- Public primary care clinic
- Private primary care clinic
- Private practice
- Other, what?....................

1. **Main workplace - size of town:**

□ village city: □ <50 thousand inhabitants □ 50-100 thousand inhabitants □ >100 thousand inhabitants

1. **Main workplace - voivodship:**

□ dolnośląskie voivodship □ łódzkie voivodship □ podkarpackie voivodship □ świętokrzyskie voivodship

□ kujawsko-pomorskie voivodship □ małopolskie voivodship □ podlaskie voivodship □ warmińsko-mazurskie

voivodship □ lubelskie voivodship □ mazowieckie voivodship □ pomorskie voivodship □ wielkopolskie

voivodship □ lubuskie voivodship □ opolskie voivodship □ śląskie voivodship □ zachodniopomorskie

voivodship

1. **Work experience (after board certification) …………………..**
2. **Average number of patients admitted per week:……………………**
3. **Average number of patients admitted weekly with AR (allergic rhinitis):………………………**
4. **Considering all your adult (over 18 years of age) AR patients, what is your most common treatment for adult patients?** *Interviewer: selects all listed answers, the interviewer does not read the possible answers.*

oral antihistamines

topical antihistamines (intranasal, conjunctivally)

intranasal glucocorticoids

leukotriene receptor antagonists

cromons

medicines that decongestant blood vessels (oral, intranasal)

systemic glucocorticoids

cholinergic receptor antagonists

combination of intranasal GCs and intranasal AH1 in one applicator

immunotherapy (SLIT, SCIT)

1. **Considering all your AR patients aged 5 to 18, what treatment do you most often give them?** *Interviewer: selects all listed answers, the interviewer does not read the possible answers.*

oral antihistamines

topical antihistamines (intranasal, conjunctivally)

intranasal glucocorticoids

leukotriene receptor antagonists

cromons

medicines that decongestant blood vessels (oral, intranasal)

systemic glucocorticoids

cholinergic receptor antagonists

combination of intranasal GCs and intranasal AH1 in one applicator

immunotherapy (SLIT, SCIT)

1. **Considering all your youngest AR patients, children up to 5 years of age, what treatment do you most often give them?** *Interviewer: selects all listed answers, the interviewer does not read the possible answers.*

oral antihistamines

topical antihistamines (intranasal, conjunctivally)

intranasal glucocorticoids

leukotriene receptor antagonists

cromons

medicines that decongestant blood vessels (oral, intranasal)

systemic glucocorticoids

cholinergic receptor antagonists

combination of intranasal GCs and intranasal AH1 in one applicator

immunotherapy (SLIT, SCIT)

1. **Please indicate what factors you take into account when choosing a drug for AR.** *I: possibility of giving multiple answers, the interviewer selects all the answers spontaneously mentioned by the doctor, he does not read the list of possible answers.*

- Efficiency
- Drug status (OTC vs RX)
- The duration of the drug's presence on the market
- Several forms of the drug adapted to the needs of different patients
- Drug dosage
- Own good experience with the drug
- Patient's age
- Drug price
- Refund / no refund of the drug
- Occurrence of adverse events
- Sedative effect
- Interactions with other drugs
- Clinical trial results
- Medical literature regarding a given drug
- Other, what?..........................

1. **Now, please provide the 3 most important factors for you when choosing a drug.** *Interviewer: enter the selection factors given by the doctor in the order (where 1 means the factor mentioned first, etc.)*
2. ………………….
3. …………………
4. …………………
5. **Which route of drug administration is preferred by patients?** *The interviewer: selects the spontaneous answer indicated by the doctor, the interviewer does not read out the possible answers.*

- oral
- intranasal
- conjunctival
- subcutaneous
- sublingual
- other, what?

1. **Which route of drug administration is preferred by patients?** *The interviewer: selects the spontaneous answer indicated by the doctor, the interviewer does not read out the possible answers.*

□ oral in solution

□ oral in tablets that dissolve in the mouth without drinking water

1. **In your opinion, how much is a patient willing to pay for monthly AR therapy, depending on its form?** *Interviewer: read the forms of AR: mild, moderate and severe and write down the amount given by the doctor for each of them.*

- Mild……………………..
- Moderate……………
- Severe………………………
- Difficult to say

1. **Does the patient choose a cheaper drug for 1 month or a more expensive drug that will be enough for 2 months of therapy?** *Interviewer: read the possible answers: cheaper for a month and more expensive for two months, mark the answer provided by the doctor.*

- Less for month
- More for two months
- Difficult to say

1. **In your opinion, how much is a patient willing to spend per month on all medications used (for all his/her ailments)?** *Interviewer: enter the amount given by the doctor*

**…………………….**

1. **What sources of information do you prefer?** *The interviewer: writes down all answers given spontaneously by the doctor, does not read the answers from the list.*

- e-mailings/newsletters
- SMSes
- MMSes
- postal items
- leaflets/printed brochures
- visits of medical representatives
- telemarketing
- information at conferences/trainings
- training with educational points
- video lectures with experts
- social media (facebook, twitter, instagram)
- podcasts with experts
- other, what?.....
- difficult to say

1. **Now, please provide the 3 most important sources of information for you.** *Interviewer: enter the sources of information given by the doctor in the order (where 1 means the source of information listed as the first, etc.)*
2. ………………….
3. …………………
4. …………………
